# Analysis of the Financing of Russian Health Care over the Past 100 Years

**DOI:** 10.3390/ijerph16101848

**Published:** 2019-05-24

**Authors:** Vladimir Reshetnikov, Evgeny Arsentyev, Sergey Bolevich, Yuriy Timofeyev, Mihajlo Jakovljević

**Affiliations:** 1 Department of Public Health and Healthcare, First Moscow State Medical University of the Ministry of Health of the Russian Federation (Sechenov University), 119991 Moscow, Russia; arsevgen22@gmail.com; 2 Department of Human Pathology, First Moscow State Medical University of the Ministry of Health of the Russian Federation (Sechenov University), 119991 Moscow, Russia; bolevich2011@yandex.ru; 3 Faculty of Business and Management, National Research University Higher Schools of Economics, 101000 Moscow, Russia; y.timofeyev@hse.ru; 4 Department of Global Health Economics and Policy, Faculty of Medical Sciences, University of Kragujevac, 34000 Kragujevac, Serbia; sidartagothama@gmail.com; 5 Division of Health Economics, Lund University, SE 220 07 Lund, Sweden

The evolution of epidemiological burden in Imperial Russia and, consecutively, the Union of Soviet Socialist Republics (USSR), took place mostly over the duration of the past century. It is very important since dozens of Eastern European and Asian nations, with similarities in life style, regardless of dominant Orthodox Christian, Sunni-Shia Islamic, or Shamanic ethno-religious patterns, share this old statehood tradition. This profound change reflected in gradual movement from communicable, infectious diseases, traumatism, and early childhood and maternal mortality towards chronic non-communicable diseases [1]. To some extent, these changes were accelerated by two world wars and the deep regulatory reforms of social and pension systems, together with health care provision and financing mechanisms imposed, as a result of the Bolshevik, October Revolution in 1917. This long-term evolution, particularly in the post-World War II decades, was ultimately associated with the occurrence of population aging throughout entire Northern Hemisphere [2]. It got worse in the East due to the deep Russian recession reaching the bottom in 1998 and effectively dragging all mutually dependent formerly centrally-planned economies [3]. Compared to their Western European counterparts, Russian and other Eastern European ethnicities mostly remain in a slightly earlier stage of population ageing, which is yet tangible by serious policies [4]. Since the beginning of the 21st century, bold economic recovery and growth took place, ultimately leading to successful fertility policies. The boost in total fertility levels, which was raised from 1.3 to 1.7 children per woman of reproductive age, was the highest net achievement of its kind by any European country during the second decade of the 21st century [5]. All of these complex historical changes following the business cycles [6] in the capitalist free-market economies had heavily reflected the ability of the Russian Federation and its predecessor states to increase investment in healthcare and provide equitable and affordable medical care to its citizens [7]. Therefore, this paper attempts to look at the inner legislative evolution of health financing in Russia over the last 100 years.

At the beginning of the 20th century, a progressive form of medical care for the population—Zemstvo district medicine—was embodied in the Russian Empire. However, a significant drawback of rural medicine was its extremely high cost. At the end of the 19th and the beginning of the 20th century, most Zemstvos spent between 25% and 40% of their budget on healthcare [8]. For example, in 1887, the Cheboksary Zemstvo district spent 40.6% of its budget on medicine. The cost of Zemstvo district medicine in 1912 in 40 Zemstvo provinces accounted for 26% of the total Zemstvo district budget [9].

After the October Revolution of 1917, the Bolshevik government made a decision about concentrating the entire healthcare management of the country in a single, competent body that would be under their full control. In addition, in 1918, in the Russian Soviet Federative Socialist Republic (RSFSR), the first nationwide medical delivery system was created [10]. The People’s Commissariat of Health was established and was a state body responsible for all the country’s health care. The first Commissar of Health was Nikolay Andreyevich Semashko [11]. 

Figure 1 shows government spending on the health care system as a percentage of the country’s total budget for the RSFSR in 1918–1921 and for the USSR in 1922–1989.

After the legalization of the central health authority of the republic, the government began the construction of a unified state system of health care financing. Until 1919, there were two main sources of health care financing in the country: the state-governed one and, superior to it, an insurance-based system, which was funded by employers’ tax contributions. Thus, for the final implementation of a single, Soviet, medicine, it was necessary to eliminate “insurance medicine”. Despite the resistance of insurance companies, in February 1919, the Council of People’s Commissars approved the document “On the transfer of the entire medical part of the former health insurance funds to the People’s Commissar of Health”, and liquidated health insurance companies.

Nevertheless, even after the acquisition of insurance resources, the funds for health care were still not sufficient. Moreover, during the period of the new economic policy, the Bolshevik government decided to revive the elements of insurance-based medical care, but within the framework of the social insurance system. In 1921–1923, the employers’ insurance premiums were determined for certain types of social insurance, including the medical insurance premium of 5.5%–7% of the wage fund. The exact values of these premiums were dependent on the type of occupational hazards [11].

However, insurance medicine was already an integral part of the unified health care system, which made it possible to accumulate additional funds to finance health care (through insurance funds formed from employers’ contributions). The contributions to the All-Union Fund were transferred from the central social insurance authorities directly to the People’s Commissariat of Health [12,13].

During this period, the state insurance model of the organization of health care actually operated. Nevertheless, there was still not enough money for health care.

Since 1928, after the start of the implementation of the five-year plans for the development of the national economy of the USSR, social policy faded into the background: the health care system was financed according to the residual principle. Due to the economic growth after the beginning of the first five-year plan, under the conditions of a shortage of resources, there was an increasing gap between the economic and social aspects of development. With a significant increase of investment in the industrial sector, the portion of expenditures allocated toward the social sphere and health care in particular decreased. Most of the medical institutions were transferred from the state to the local budget, which was not sufficient everywhere. This circumstance led to the shutdown of a number of medical institutions and the introduction of paid medical services. However, soon the 3rd All-Russian Congress of Health Departments proclaimed the inviolability of the basic principles of health care: namely, its state-funded and free-of-charge basis. This important change ultimately led to the Semashko system, delivering the first-ever universal health coverage nationwide, to all of its citizens, regardless of their household income, at the basic medical technology level of that time. This contribution is recognized in health economics literature [7]. 

In the 1930s, the residual principle of health care financing remained in the republic: allocations for health care were decreasing. However, in 1934, elements of insurance medicine were completely abolished, and allocations for medical assistance to the insured were included into the general health budget. Financing of medical care was carried out exclusively at the expense of state budget funds. This model of healthcare financing existed until 1989 [14].

It is fair to say that “free” health care in the USSR (like other social funds) was provided by direct underpayments for labor and deductions from wages in the form of taxes. Thus, the state acted as a mediator, performing the functions of redistribution, transferring of funds from employment income to those citizens who could not provide themselves with a subsistence minimum [15].

After the outbreak of World War II, allocations for health care steadily declined due to the need to develop the military industry. A particularly sharp decline in the financing of health care occurred in 1941, after the USSR entered World War II. In 1941, healthcare accounted for only 3.6% of the total budget of the USSR. Only by 1948, it became possible to achieve pre-war levels of spending on healthcare: 5.3% of the total budget.

However, since 1950, due to the rearmament of the army, as well as the indirect participation of the USSR in the Korean War, healthcare expenditure again decreased, reaching 4.6% of the total budget in 1953.

In the period of the Khrushchev’s “*thaw*” in the USSR, the amount of government expenditure directed toward health care began to grow. By the early 1960s, healthcare expenditure reached 6%–6.5% of the total budget of the USSR.

Between 1965 and 1980, the real level of healthcare spending increased. Low inflation led to a substantial increase in real spending. However, this real growth did not keep pace with the general growth of the economy and social spending. The portion of health care in the state budget fell from 6.5% in 1965 to 4.5% by 1985. This circumstance indicated both a worsening of the economic situation and a decreased priority role of healthcare in the government’s agenda.

In the 1960s, approximately 6%–6.5% of GDP was allocated for health care, and in the last years of the USSR’s existence the level dropped to 3%–3.5%, which was significantly lower than in the countries of the Organization for Economic Cooperation and Development (OECD). (For comparison, in 1985, this rate was 6.5% and 12.9% in the United Kingdom and the United States, respectively) [16].

During the “*perestroika*” period (period of transformation), the Soviet government significantly increased total health expenditure in nominal terms, but during these years the inflation rate exceeded the increase in spending. The portion of the state budget allocated toward health care grew, reaching a value of 5.6% in 1990 (not more than 3% of GDP) [17].

Thus, since 1985, the actual level of expenditure on healthcare again steadily declined, reaching 2.6% of GDP by 1995. The situation was particularly aggravated by the difficult economic situation in the country associated with the collapse of the USSR.

Figure 2 demonstrates public healthcare expenditure in the Russian Federation. The reform of the Russian health care financing began in 1991 due to the inability of the state to provide the formerly guaranteed volume of free medical services. The German model of health care, based on the principles of medical insurance, was chosen as a guideline. This model was considered to be the most optimal for Russia, since it provided for the preservation of state control in financing (and, therefore, control of the health care system) [18].

However, the transition to the budget-insurance model of health financing did not allow for more than 3.1% of GDP to be allocated to healthcare. Only since 2005, after an increase in budget allocations, spending on healthcare reached 3.7% of GDP. Despite a certain positive trend (an increase in spending on health care to 4.3% of GDP in 2009), in 2013–2014, health financing fell again to 3.2% of GDP. In 2016, this rate was 3.6% of GDP. Thus, in the history of Russian healthcare, expenditure on healthcare never exceeded the Soviet level of 6–6.5%. The health system input–output ratio can be roughly observed through the relationship between total health spending and core efficiency indicators, such as life expectancy at birth. According to recent research, the Russian Federation and the surrounding countries of the Commonwealth of Independent States (CIS) are achieving bold progress, although are still lagging against Western Europe [19]. Current health system vulnerabilities also present room for improvements and hidden opportunities in the form of net gains in human longevity and improvements in other morbidity- and mortality-related outcomes to be achieved by increased investment in healthcare [20]. This fact presents a window of opportunity for the Russian Federation’s future healthcare planning strategies during the 2020s. 

## Figures and Tables

**Figure 1:**
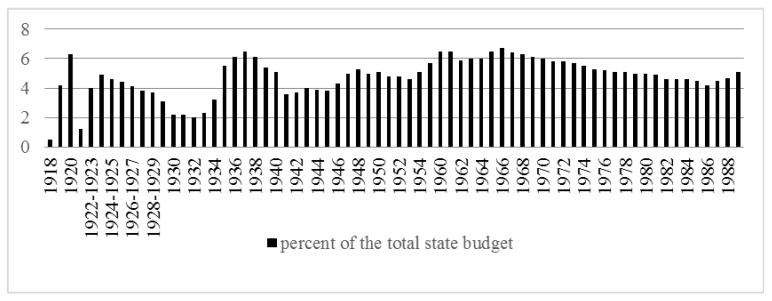
Government expenditure on health from the Russian Soviet Federative Socialist Republic (RSFSR) to the Union of Soviet Socialist Republics (USSR).

**Figure 2:**
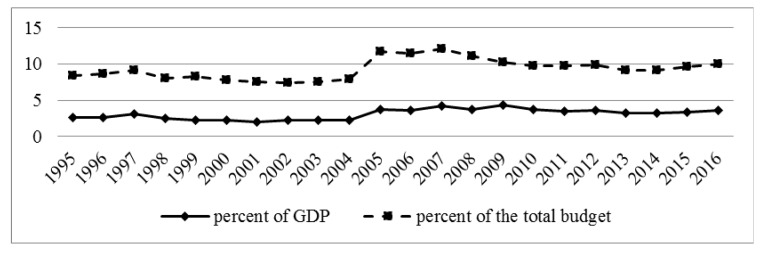
Expenditures of the budget system of the Russian Federation on the health care system (consolidated budget of the Russian Federation and budgets of state extra-budgetary funds). Note: Since 2005, data on the consolidated budget of the Russian Federation are provided taking into account the budgets of state extra-budgetary funds. From 1995 to 2010, the cost of physical culture is taken into account. Since 2011, only expenditure on healthcare has been presented. Budgets of state extra-budgetary funds include compulsory health insurance. Thus, the compulsory insurance contributions of all Russian employers are also included in the official statistical report of public expenditure on health.
